# Anomalous Surplus Energy Transfer Observed with Multiple FRET Acceptors

**DOI:** 10.1371/journal.pone.0008031

**Published:** 2009-11-25

**Authors:** Srinagesh V. Koushik, Paul S. Blank, Steven S. Vogel

**Affiliations:** 1 National Institute on Alcohol Abuse and Alcoholism, National Institutes of Health, Rockville, Maryland, United States of America; 2 Eunice Kennedy Shriver National Institute of Child Health and Human Development, National Institutes of Health, Bethesda, Maryland, United States of America; Mount Sinai School of Medicine, United States of America

## Abstract

**Background:**

Förster resonance energy transfer (FRET) is a mechanism where energy is transferred from an excited donor fluorophore to adjacent chromophores via non-radiative dipole-dipole interactions. FRET theory primarily considers the interactions of a single donor-acceptor pair. Unfortunately, it is rarely known if only a single acceptor is present in a molecular complex. Thus, the use of FRET as a tool for measuring protein-protein interactions inside living cells requires an understanding of how FRET changes with multiple acceptors. When multiple FRET acceptors are present it is assumed that a quantum of energy is either released from the donor, or transferred *in toto* to only one of the acceptors present. The rate of energy transfer between the donor and a specific acceptor (*k_D→A_*) can be measured in the absence of other acceptors, and these individual FRET transfer rates can be used to predict the ensemble FRET efficiency using a simple kinetic model where the sum of all FRET transfer rates is divided by the sum of all radiative and non-radiative transfer rates.

**Methodology/Principal Findings:**

The generality of this approach was tested by measuring the ensemble FRET efficiency in two constructs, each containing a single fluorescent-protein donor (Cerulean) and either two or three FRET acceptors (Venus). FRET transfer rates between individual donor-acceptor pairs within these constructs were calculated from FRET efficiencies measured after systematically introducing point mutations to eliminate all other acceptors. We find that the amount of energy transfer observed in constructs having multiple acceptors is significantly greater than the FRET efficiency predicted from the sum of the individual donor to acceptor transfer rates.

**Conclusions/Significance:**

We conclude that either an additional energy transfer pathway exists when multiple acceptors are present, or that a theoretical assumption on which the kinetic model prediction is based is incorrect.

## Introduction

Förster resonance energy transfer (FRET) is a near-field mechanism by which energy is transferred from a donor fluorophore to an adjacent chromophore via nonradiative dipole-dipole interactions [1], [2], [3], [4], [5], [6]. FRET theory is applicable only to fluorophores that have very weak coupling [7], and primarily considers the interactions of a single donor-acceptor pair when they are separated by between 1–10 nm [5], [6], but can be expanded to cover the situation when more than one acceptor is present if one assumes that a donor interacts with each acceptor *independently*. Even though the independence of parallel-acting fluorescence deactivation pathways is one of the cornerstones on which spectroscopy is built [5], the validity of this assumption when applied to energy transfer in the near-field has not been directly tested. Clearly, the use of FRET as a comprehensive tool for measuring protein-protein interactions requires an understanding of how FRET values change when more than one acceptor is present [6], [8].

The FRET efficiency of a donor–acceptor pair is defined as *the fraction of the photon energy absorbed by a fluorescent molecule that is transferred to an acceptor* [*4*] *,* [*5*] *,* [*6*]. If *k_D→A_* is the rate of energy transfer from a donor to an acceptor in the presence of a single acceptor, and τ*_D_* is the fluorescence lifetime of the donor fluorophore in the absence of acceptors, then E, the FRET efficiency is [4], [6]:
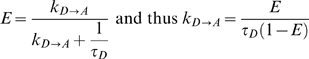



This kinetic formalism has been modified to calculate the FRET efficiency between a donor and multiple acceptors. For example, the FRET efficiency when two acceptors are present is thought to be [4], [6]:
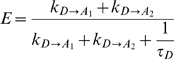
(1)


This model allows energy from a donor to be transferred discretely to two acceptors, and *assumes* that each behaves independently in parallel-acting deactivation paths. The general form for equation 1 when *i* acceptors are present is:
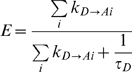
(2)


## Results

**Figure 1:**
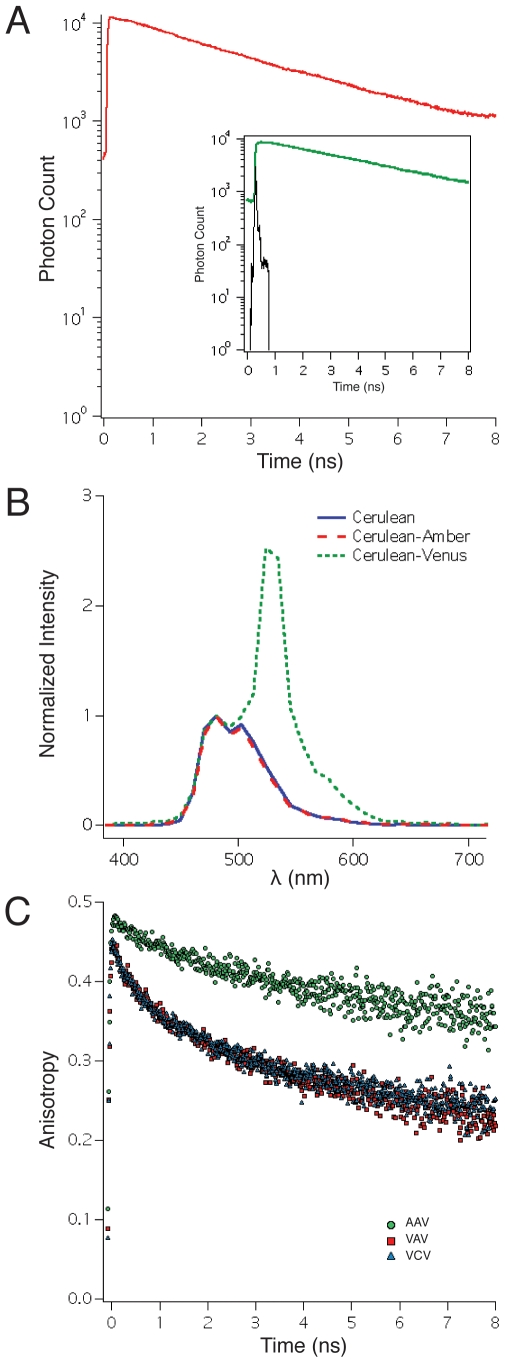
The Lifetime of Cerulean when flanked by two Amber molecules. A. The fluorescence lifetime of Cerulean in the ACA construct was measured by time-correlated single photon counting (RED trace is the mean of traces observed in 5 different cells expressing ACA). Fluorescein at pH 10 was used as a lifetime standard to validate the accuracy of our instrumentation and its fluorescence lifetime decay is depicted in the inset (GREEN trace, mean of 3 measurements). The instrument response function of our FLIM system is also depicted in the inset (BLACK trace). B. The normalized emission spectrum of cells transfected with either Cerulean (BLUE trace), Cerulean attached to Amber (RED dashed trace), or Cerulean attached to Venus (GREEN dotted trace). Each trace is the average of 3 cells, and all samples were excited with two-photon excitation at 820 nm. Traces were normalized to the peak of the Cerulean emission at 481 nm. C. The time resolved fluorescence anisotropy decay of Venus in cells transfected with AAV (GREEN circles), VAV (RED squares), or VCV (BLUE triangles). Each point is the mean of 10 cells excited at 950 nm.

To test the generality of the kinetic model for FRET with multiple acceptors we engineered a set of genetic constructs composed of different mixtures and arrangements of three spectral variants of Green Fluorescent Protein (FP), using Cerulean [9] (as a FRET donor), Venus [10] (as the acceptor), and *Amber* [11] a Venus-“like” molecule that has a point mutation preventing fluorophore formation and presumably it can't act as a FRET acceptor. Amber-Cerulean-Amber (ACA) was generated to measure the fluorescence lifetime of Cerulean in the absence of FRET when attached to FP's on both its C- and N-termini. Cerulean in ACA had a lifetime of 2.95±0.02 ns (mean±SEM, n = 5 cells) when measured in living cells by time correlated single photon counting (TCSPC) [12] (Fig. 1A). In figure 1B we compare the emission spectrum of Cerulean, Cerulean attached to Amber (C5A), and Cerulean attached to Venus (C5V) when excited with 820 nm two-photon excitation. The emission spectrum of C5A was indistinguishable from the spectrum of Cerulean and both were different than the emission spectrum of C5V indicating that Amber is not fluorescent. Time-resolved fluorescence anisotropy decay of a fluorescent protein in a complex is sensitive to the mass and shape of the protein as well as to the number of fluorophores and rate of energy migration in the complex [13]. Venus fluorescence was excited with 950 nm two-photon excitation and the Venus time-resolved fluorescence anisotropy decay was measured for three structurally related constructs, Amber-Amber-Venus (AAV), Venus-Cerulean-Venus (VCV) and Venus-Amber-Venus (VAV) (Fig. 1C). Because the AAV construct has only a single fluorophore its anisotropy decay curve will reflect the rotation of the Venus fluorophore in this complex as a function of mass and shape. In contrast, both VCV and VAV have two Venus fluorophores attached by either a Cerulean or Amber molecule. Thus, in addition to depolarization caused by molecular rotation, these constructs should also have a fast anisotropy decay component due to homo-FRET. If the structure of Amber and Cerulean are essentially the same, the separation distance between the two Venus fluorophores in VCV and VAV should also be the same and therefore the homo-FRET transfer between these fluorophores should have similar rates. The anisotropy decay curve of VCV and VAV were virtually identical consistent with Amber having the same β-barrel folding pattern of Cerulean [14]. Furthermore the slow decay component observed in AAV was similar to the slow decay component of both VCV and VAV indicating again that Amber has a similar folding structure as Cerulean [14] and Venus [15]. Thus we conclude that Amber is not a dark absorber, is not fluorescent, but has a similar three dimensional structure as Cerulean and Venus.

**Figure 2:**
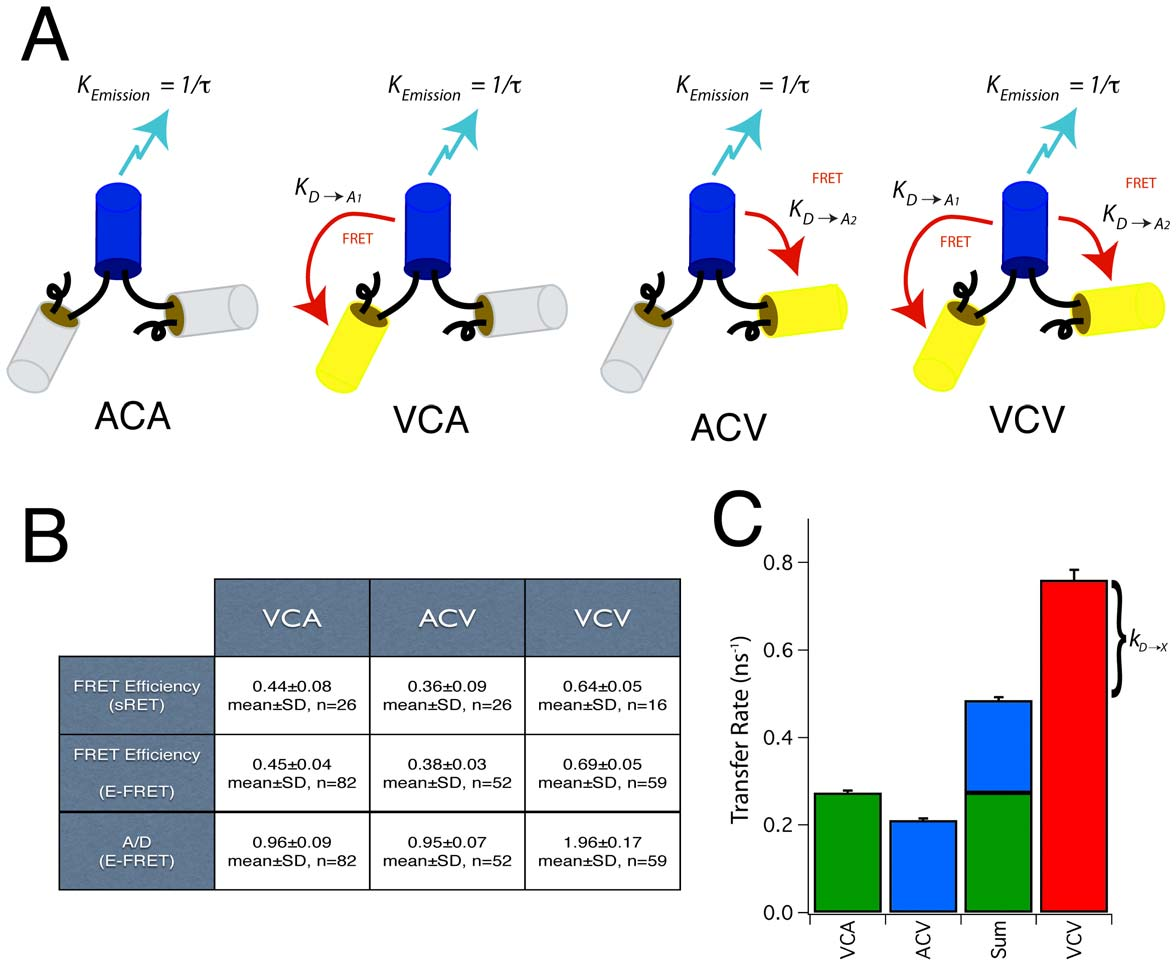
Predicting the FRET efficiency of a construct with two acceptors. A. Constructs used to study the effects of having two acceptors in a FRET complex. Blue “cans” depict the Cerulean donor, Yellow depicts Venus acceptors, and gray depicts Amber, which has a single point mutation in Venus that prevents it from forming a fluorophore. Arrows leading away from Cerulean represent both radiative and non-radiative emission pathways. Blue arrows show radiative emission when Cerulean emits a photon. Red arrows depict non-radiative pathways for releasing excitation energy involving FRET. B. Table comparing the FRET efficiencies of VCA, ACV, and VCV measured by either sRET or E-FRET, as well as the measured acceptor to donor ratio (A/D) for each construct. **C.** Energy transfer rates and their propagated error were calculated from the measured Cerulean lifetime of ACA and the FRET efficiencies measured by E-FRET of individual constructs. The Sum column is the arithmetic sum of the VCA and ACV transfer rates with propagated error and by theory should equal the transfer rate measured for VCV.

Amber-Cerulean-Venus (ACV) and Venus-Cerulean-Amber (VCA) are structurally related constructs generated to measure the FRET efficiency (and transfer rate) between Cerulean and either a C- or N-terminal Venus (Fig. 2). In ACV the linker separating Cerulean and Venus is 6 amino acids while in VCA it is only 5. They had FRET efficiencies of 0.36±0.09 (mean±SD, n = 26) and 0.44±0.08 (n = 26) respectively, as determined by sRET analysis [16] of two-photon excitation spectral images [17]. The energy transfer rates of ACV and VCA were calculated using their FRET efficiencies and the Cerulean lifetime in ACA, and were found to be 0.19±0.01 and 0.26±0.02 ns^−1^ (mean±propagated SEM). VCV, a FRET construct with 1 donor and 2 acceptors, had a transfer efficiency of 0.64±0.05 (n = 16). This value was similar to FRET efficiencies measured for VCV in a previous study [16] (0.70±0.06 by sRET, 0.65±0.03 by FLIM-FRET), but was larger, and statistically different from the value predicted using ACV and VCA transfer rates in equation 1 (0.58±0.01, mean±propagated SEM, p<0.01).

While the difference between the measured VCV FRET efficiency and the transfer efficiency predicted by the kinetic model was statistically different, it was also relatively small (6%). Accordingly, we wanted to corroborate this observation using a different FRET method that did not rely on either two-photon or laser excitation. E-FRET [18], [19], a method of measuring FRET efficiency based on acceptor desensitization was selected because it uses an arc lamp as a one-photon excitation source. An additional attraction of the E-FRET approach is that it can accurately measure the acceptor to donor ratio [18], [19]. This then could be used to test if Cerulean and Venus molecules generated their fluorophores, and if they were present in the stoichiometry predicted by the sequence of a specific construct. E-FRET analysis revealed that ACV had a FRET efficiency of 0.38±0.03 (mean±SD, n = 52) and was expressed at the expected acceptor to donor ratio of 1 for a molecule with one acceptor and one donor (0.95±0.07). VCA had a FRET efficiency of 0.45±0.04 (n = 82) and an acceptor to donor ratio of 0.96±0.09. The energy transfer rates calculated using these FRET efficiency values were 0.21±0.00 (Note that errors of 0.00 indicate a truncated error that was ≤0.005.) and 0.27±0.00 ns^−1^ (mean±propagated SEM). E-FRET analysis indicated that VCV had an acceptor to donor ratio of 1.96±0.17 (n = 59) as expected for a molecule with two acceptors and one donor. The measured FRET efficiency for VCV was 0.69±0.01 (mean±SEM). Again. this value was larger than the value predicted using equation 1 (0.59±0.01, mean±propagated SEM). The differences between the predicted and measured values is 0.10±0.02 (difference±99% confidence). As the difference does not include zero at the 99% confidence level, we reject the hypothesis that the experimentally measured values for VCV agree with the predicted values derived from the individual FRET efficiencies.

**Figure 3:**
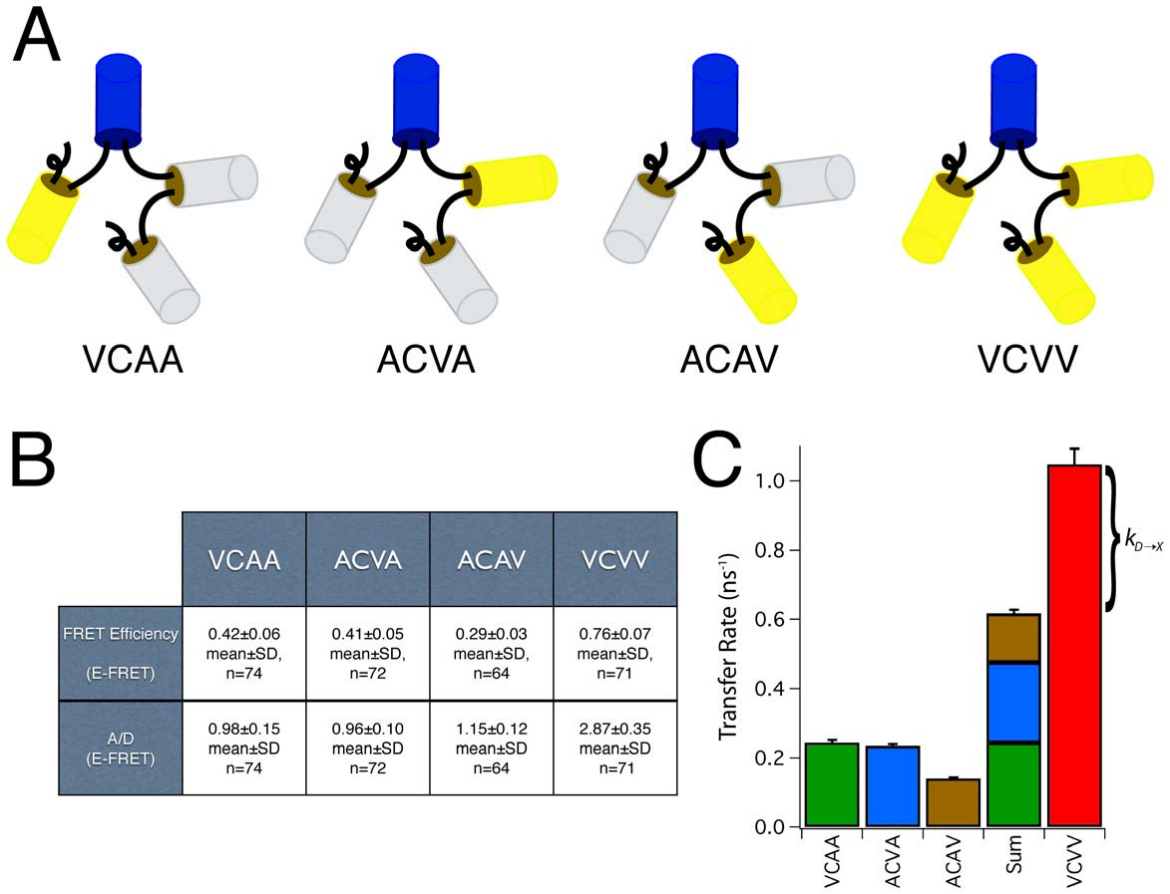
Predicting the FRET efficiency of a construct with three acceptors. A. Constructs used to study the effects of having three acceptors in a FRET complex. Blue “cans” depict the Cerulean donor, Yellow depicts Venus acceptors, and gray depicts Amber. B. Table showing the FRET efficiencies of VCAA, ACVA, ACAV and VCVV measured by E-FRET, as well as the measured acceptor to donor ratio (A/D) for each construct. C. Energy transfer rates and their propagated error were calculated from the measured Cerulean lifetime of ACA and the FRET efficiencies measured by E-FRET. The Sum column is the arithmetic sum of the VCAA, ACVA and ACAV transfer rates with propagated error, and by theory should equal the transfer rate measured for VCVV.

To test if the observed excess energy transfer was unique to the VCV construct with two acceptors, or represented a more general case when multiple acceptors are present, a set of constructs was generated to investigate FRET between a single donor and 3 acceptors (Fig. 3). Amber-Cerulean-Venus-Amber (ACVA) had a FRET efficiency of 0.41±0.05 (mean±SD, n = 72) and an acceptor to donor ratio of 0.96±0.10 as measured by E-FRET. Venus-Cerulean-Amber-Amber (VCAA) had a FRET efficiency of 0.42±0.06 (n = 74) and an acceptor to donor ratio of 0.98±0.15, and Amber-Cerulean-Amber-Venus (ACAV) had a FRET efficiency of 0.29±0.03 (n = 64) and an acceptor to donor ratio of 1.15±0.12. The calculated donor to acceptor transfer rates for these three donor-acceptor pairs was 0.23±0.00, 0.24±0.01, and 0.14±0.00 ns^−1^ respectively (mean±propagated SEM), and predicted an ensemble FRET efficiency of 0.65±0.01 (mean±propagated SEM) for a Venus-Cerulean-Venus-Venus (VCVV) construct. The FRET efficiency measured for VCVV was 0.76±0.01 (mean±SEM, n = 71) and had an acceptor to donor ratio of 2.87±0.35 (mean±SD) as expected for a complex with one donor and three acceptors. The differences between the prediction and measured values for VCVV is 0.11±0.02 (difference±99% confidence). As the difference does not include zero at the 99% confidence level, we reject the hypothesis that the experimentally measured values for VCVV agree with the predicted values derived from the individual FRET efficiencies.

**Figure 4:**
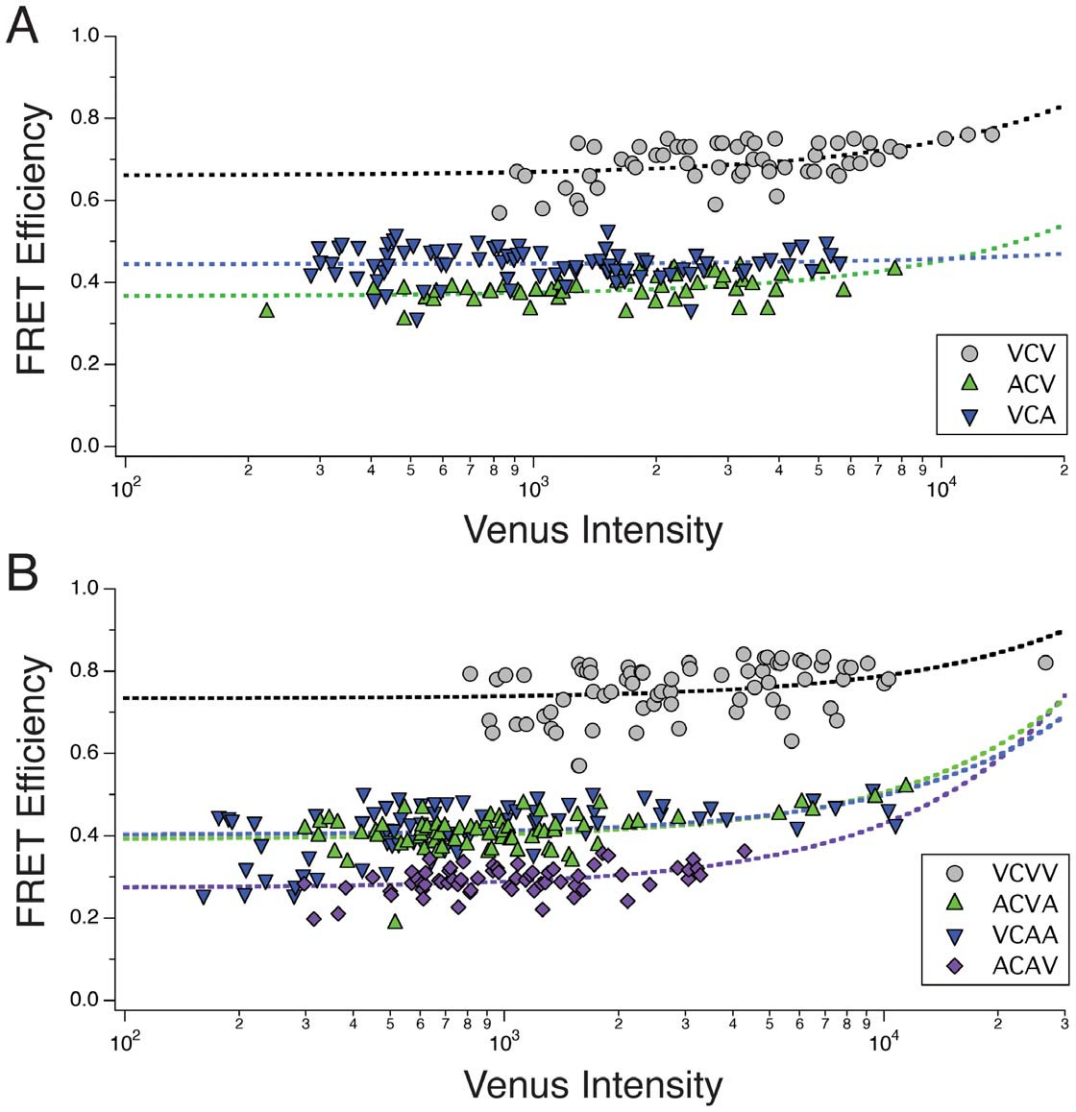
Estimating the intramolecular FRET efficiency. E-FRET was used to measure the FRET efficiencies and the acceptor intensity (Venus) for each cell expressing members of the VCV series (A; VCV, ACV, and VCA) or the VCVV series (B; VCVV, ACVA, VCAA, ACAV), and these values are plotted as a function of the exposure normalized Venus intensity. Dashed lines indicates linear regression fitting for each data set to calculate the y-intercept as an estimate of the amount of intramolecular FRET in the absence of intermolecular FRET. Note that the data is plotted on a semi-log scale to more easily reveal the full range of the Venus intensity, but in so doing make linear plots appear curved.

*Intra-*molecular FRET is energy transfer that occurs between a donor and acceptors within a molecular complex, while *inter*-molecular FRET is energy transfer that can occur between a donor in one complex, and an acceptor in another as a result of molecular crowding. If intermolecular FRET occurs and is not accounted for, a measured ensemble FRET efficiency may be an overestimation of the true intramolecular FRET efficiency. For covalently linked cytoplasmic complexes, like those in the VCV and VCVV series, significant levels of intermolecular FRET should only occur if these constructs are expressed at a very high concentration (in the mM range [8]). Experimentally, intermolecular FRET can be detected as an increase in the FRET efficiency with increased acceptor concentration. If detected, the true intramolecular FRET efficiency can be estimated from the extrapolated FRET efficiency value at infinitely dilute acceptor concentrations; the y-intercept. In E-FRET experiments, in addition to measuring FRET efficiency and the acceptor to donor ratio, the intensity of the directly excited acceptor, Venus, normalized to exposure time, is also measured. The intensity of the directly excited acceptor should be proportional to the acceptor concentration. To test and control for any intermolecular FRET, the measured FRET efficiencies of each cell that expressed constructs in either the VCV series (Fig 4A) or the VCVV series (Fig 4B) were plotted as a function of the acceptor concentration. Only minor increases in FRET efficiency were observed with increasing Venus intensity over a 1–2 order of magnitude range. Nonetheless, each data set was well fit by a linear regression, and therefore the y-intercepts were used to estimate extrapolated FRET efficiencies ± the 95% confidence levels (VCA = 0.45±0.01, ACV = 0.37±0.01, VCV = 0.66±0.02, VCAA = 0.40±0.02, ACVA = 0.39±0.01, ACAV = 0.27±0.01, VCVV = 0.73±0.02). These values were taken as the intramolecular FRET efficiency, free from intermolecular FRET. As expected, the extrapolated FRET efficiencies for any particular construct was either statistically indistinguishable, or only slightly reduced from the FRET efficiencies measured previously as the mean of the ensemble. Regardless, using these extrapolated FRET efficiencies, the kinetic model predicts a VCV FRET efficiency of 0.58±0.01, and a VCVV FRET efficiency of 0.63±0.02 (FRET efficiency±propagated 95% confidence level). The differences between the kinetic model prediction and the measured values for VCV was 0.08±0.03, and for VCVV was 0.10±0.03 (difference ±99% confidence). As these differences do not include zero at the 99% confidence level, we reject the hypothesis that the experimentally measured values for VCV and VCVV, even when adjusted for intermolecular FRET, agree with the predicted values derived from the individual FRET efficiencies.

## Discussion

The objective of this study was to experimentally test the generality of using the sum of individual donor-acceptor FRET transfer rates (*k_D→A_*) to predict the ensemble FRET efficiency of a complex of a donor with multiple acceptors (equation 2). We specifically wanted to perform this test using spectral variants of GFP in living cells as this is a common approach used to study protein-protein interactions under physiological conditions. This kinetic formalism consistently failed to predict the measured ensemble FRET efficiency. This was observed with both one- and two-photon excitation, with both laser and arc lamp excitation, and using two different methods for measuring FRET, one based on spectral imaging [16], and one based on accepter desensitization [18], [19]. What is the reason for the discrepancy between the ensemble FRET efficiencies measured when multiple acceptors are present as compared to the values predicted by theory? It is worth considering six possible explanations: 1. The fluorescence lifetime of Cerulean in the absence of acceptors that was used for our calculations is inaccurate, 2. Amber is behaving aberrantly, 3. Cerulean and/or Venus have different folding efficiencies in different constructs, 4. The additional energy transfer results from intermolecular FRET, 5. An additional occult energy transfer pathway from the donor exists, and must be accounted for, and 6. One of the theoretical assumptions on which the kinetic formalism is based is wrong.

The lifetime of Cerulean in ACA was found to be 2.95±0.02 ns (mean±SEM, n = 5 cells). This lifetime was measured on a FLIM system that had an excitation laser pulse width of less than 200 fs, and was acquired using a microchannel plate photomultiplier tube (Hammamatsu R3809U-52) that had a measured system response function of <40 ps (FWHM; Fig. 1A). The lifetime measurement was performed on five replicate cells transfected with DNA encoding ACA. Each decay curve had a minimum of 5000 peak photon counts, and was fit using a double exponential decay model deconvolved from the measured instrument response function for added precision. Furthermore, the accuracy of our TCSPC system was validated using a NIST certified Fluorescein standard (Invitrogen) at pH 10, and yielded a lifetime value of 4.08±0.00, (mean±SD, n = 3; Fig. 1A). The expected fluorescence lifetime of Fluorescein at pH 10 is 4.1 ns [11]. Most importantly, the lifetime of ACA was similar to the previously measured lifetime of Cerulean when expressed alone in cells (2.94±0.11 ns, mean±SD) [11] indicating that Cerulean's lifetime did not noticeably change when Amber molecules were added to both its C- and N-termini. Thus, it is highly unlikely that the measured ACA lifetime was off by more than 100 ps, and the actual Cerulean lifetime standard error of the mean in ACA was 20 ps. Moreover, it can be shown that the lifetime of a donor, in the absence of acceptors, is not required to predict the ensemble FRET efficiency; it can be calculated using only the FRET efficiencies of individual donor-acceptor transfer efficiencies (see Materials and Methods *Kinetic Model predictions and error propagation*). Consequently, errors in the lifetime of ACA cannot account for this discrepancy.

In this study a single point mutation in the sequence that forms Venus's fluorophore, Y_67_C, was used to form Amber, essentially a Venus protein lacking its internal chromophore. Spectroscopy was used to show that the addition of Amber to a Cerulean does not alter Ceruleans fluorescence emission profile, confirming the absence of the Venus fluorophore in Amber (Fig. 1B). In a previous study we demonstrated that the ligation of Amber to only the C-terminus of Cerulean did subtly alter its lifetime (by ∼200 ps). This result was different than the result reported above for ACA. Nonetheless this small shift in Cerulean lifetime did not change as a function of linker length [11], as was observed in homologous constructs where Venus was attached to Cerulean's C-terminus. In that case, the Cerulean lifetime was dramatically reduced by at least 1 ns, and consistently became even shorter as the number of amino acids in the linker separating the two fluorophores was reduced from 32 to 17 and then to 5 [11]. Accordingly, it was concluded that the Amber point mutation did not create a new chromophore that could act as a dark absorber for Cerulean fluorescence.

It is assumed in this study that the systematic introduction of Amber point mutations in VCV and in VCVV did not alter the separation distance or dipole orientation factor (κ^2^) of the remaining Cerulean-Venus FRET pairs in these constructs. This seems like a reasonable assumption as Tyrosine_67_ is known to reside inside the Venus β-barrel structure, not on its surface [15]. Moreover, the difference in mass between a Tyrosine and a Cysteine is 60 g/mole, thus in the worst case scenario with two Amber substitutions, the difference in mass between the VCVV construct and the mass of ACVA, VCAA, or ACAV is only 0.1%. We also note that if the introduction of Amber was to cause a shift in the separation distance or dipole orientation factor of the remaining Cerulean-Venus FRET pairs, we would expect both positive and negative changes in the remaining FRET transfer rates. The results reported here, however, would only be possible if the introduction of the Amber mutation always resulted in a significant net decrease in the remaining FRET transfer rate (∼36% for ACV and VCA, and ∼41% for ACVA, VCAA, and ACAV). Nonetheless, to investigate this possibility further, fluorescence anisotropy decay analysis was used to compare the three dimensional structure of Amber to the known β-barrel structures of Cerulean [14] and Venus [15] (Fig. 1C). The similarity between the fluorescence anisotropy decay of VCV and VAV indicate that both the homo-FRET transfer rates between the two Venus molecules in these constructs, as well as the rotational correlation time for these fluorophores were nearly identical [13]. We conclude that the three dimensional structure of Amber is likely to be a β-barrel like Cerulean and Venus, and that it is extremely unlikely that the introduction of Amber caused a change in the separation distance or dipole orientation factor of the remaining Cerulean-Venus FRET pairs. It should also be noted that because the rotational correlation times of Venus and Cerulean are much longer than their fluorescence lifetimes [20], there will be little if any motion of these fluorophores during a steady-state FRET measurement. Thus, it is unlikely that the discrepancies observed in this study can be explained by changes in FRET pair separation distance or κ^2^ value as a result of molecular rotation between excitation and emission.

It has been suggested that a large fraction of fluorescent proteins fail to fold and form chromophores when expressed in cells [21]. If true, the interpretation of fluorescent protein FRET measurements will require accounting for the fraction of miss-folded donors and acceptors. This extreme conclusion was based on a model dependent interpretation of the absence of a single exponential decay in the analysis of the fluorescence lifetime decay of tandem fluorescent protein constructs with one donor and one acceptor. An alternative explanation for this observation, however, is that tandem fluorescent protein constructs have a distribution of separation distances between donors and acceptors rather than one discrete separation distance. This too would result in a multi-exponential decay, even if both fluorophores folded normally and efficiently. To differentiate between these possibilities, we employed E-FRET analysis [18], [19] that in addition to the ensemble FRET efficiency, also yields the acceptor to donor ratio in a cell. We found that under our culture conditions the predicted acceptor to donor ratio for a particular construct matched the ratio experimentally measured. Thus, it seems highly unlikely that a significant fraction of the fluorophores in these constructs fail to form as a result of improper protein folding.

One potential explanation for the excess energy transfer observed in cells expressing either VCV or VCVV is that the cells expressing these constructs had significantly higher levels of intermolecular FRET than observed in cells expressing the control constructs (e.g. ACV, VCA for the VCV series, and ACVA, VCAA, and ACAV for the VCVV series). In figure 4, measured FRET efficiencies were plotted as a function of acceptor concentration. Only a small increase in FRET efficiency as a function of the Venus intensity was observed over an acceptor intensity range spanning at least 1 order of magnitude for each construct. Linear regression analysis of the data set for each construct was used to estimate the intramolecular FRET efficiency in the absence of intermolecular FRET. These extrapolated intramolecular FRET efficiencies were then used to predict the VCV and VCVV FRET efficiency using the kinetic model. Even when using these adjusted FRET efficiencies, the kinetic model failed to accurately predict the measured VCV or VCVV FRET efficiencies. We conclude that the existence of intermolecular FRET in our samples, if any, is not responsible for the observed surplus energy transfer.

If an additional occult energy transfer pathway that removes excitation energy from the donor exists, the ensemble transfer efficiency would be:
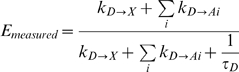



Where *k_D→X_* is the energy transfer rate of this new hypothetical pathway. Accordingly, this equation can also be used to estimate the transfer rate *k_D→X_* (and transfer efficiency, *E_X_*) of this additional energy transfer pathway if the ensemble FRET efficiency and the individual FRET transfer rates are known. For the VCV E-FRET experiment with two Venus acceptors described above using the mean E-FRET efficiency values from Figs 2B and 3B we calculate that *E_X_* = 0.45±0.02 (*k_D→X_* = 0.28±0.02 ns^−1^, mean±propagated SEM), and for VCVV with three Venus acceptors we calculate that *E_X_* = 0.56±0.03 (*k_D→X_* = 0.43±0.05 ns^−1^). Conceptually, *k_D→X_* for VCV can be thought of as the difference between the VCV and Sum transfer rate in figure 2C, and *k_D→X_* for VCVV can be thought of as the difference between the VCVV and the Sum transfer rate in figure 3C. These transfer rates are not the same. Surprisingly, *k_D→X_* increased with the number of acceptors, and appeared to scale with the number of Venus molecules present in a construct (∼0.14 ns^−1^/Venus acceptor). This suggests that the additional pathway, if it exists, physically involves interactions between Cerulean and these Venus molecules. It is also worth noting that the *k_D→X_* transfer rate predicted for VCVV (0.43 ns^−1^) is considerably faster than the three FRET transfer rates measured between the Cerulean donor and the individual Venus acceptors (0.23, 0.24, and 0.14 ns^−1^). One way to explain the discrepancy between the measured VCVV FRET efficiency of 0.76 with the FRET efficiency predicted by the kinetic model (0.65) is to assume that the FRET efficiency measurements of ACVA, VCAA, and ACAV each underestimated the ‘true’ FRET efficiency by approximately 0.12–0.13. In terms of transfer rates we would have had to underestimate them by 41%. This seems unlikely as we have previously shown that E-FRET measurements were statistically indistinguishable from FLIM-FRET measurements [11], and that both methods could differentiate changes as small as 5% in FRET efficiencies [11]. Furthermore, the E-FRET system used in this study was calibrated with FRET reference standards [11], [19], and the Acceptor/Donor ratio measured simultaneously by E-FRET for all of these constructs were correct to the closest integer (1 for ACVA, VCAA, ACAV, 3 for VCVV). We conclude that in VCVV the dominant energy transfer pathway from an excited Cerulean fluorophore is not radiative emission or classical FRET transfer to any of the three attached Venus molecules, rather it results from a poorly understood additional energy transfer pathway.

What kind of physical process can account for energy transfer at this high rate? Both Cerulean and Venus are fluorophores trapped in a β-barrel protein shell. Thus, it is unlikely that a Dexter electron exchange mechanism is possible because the closest that these fluorophores can approach one another is ∼2–3 nm [7], [22]. Similarly, at these separation distances, and particularly at physiologically relevant temperatures [23], it also seems unlikely that fluorophores can be strongly coupled [7]. It is also unlikely that this additional pathway results from the presence of an endogenous cellular quencher as: 1. Cerulean's lifetime when attached to other proteins at both its C- and N-terminus in the absence of acceptors (ACA) was also measured in living cells; thus, if quenchers were present in these cells, they would be accounted for in our calculations, 2. Cerulean's lifetime in cells [11] was similar to the lifetime of purified Cerulean in buffers whose refractive index was matched to that of cytoplasm [24]; thus it is unlikely that hypothetical endogenous quenchers significantly altered our ACA lifetime measurement, 3. Both Cerulean and Venus are not quenched by low molecular weight quenchers such as acrylamide or potassium iodide [20], and 4. As mentioned above, the energy transfer rate *k_D→X_* increased with the number of Venus acceptors present in a construct. This suggests that the Venus chromophore is the ‘quencher’ responsible for this excess energy transfer. While we cannot rule out an additional energy transfer mechanism from Cerulean facilitated by physical contact between the walls of adjacent β-barrel protein shells, such a mechanism would still require the presence of Venus chromophores inside the β-barrel, as the lifetime of Cerulean in ACA was similar to the lifetime of Cerulean alone, and both were much longer than the previously measured fluorescence lifetime of Cerulean in VCV [16]. Furthermore, such a mechanism seems unlikely as all Cerulean, Venus, and Amber molecules used in this study contained the A_206_K mutation that prevents fluorescent protein aggregation [25].

Without a compelling explanation for the excess energy transfer observed when multiple Venus acceptors were present, we must conclude either that there exists an inexplicable new transfer mechanism from a Cerulean donor to Venus acceptors, or consider the possibility that one of our assumptions for applying the kinetic formalism to calculate the ensemble FRET efficiency of either VCV or VCVV is wrong. The assumptions involved in FRET energy transfer have been clearly explained by Förster [1]. Moreover, many of the predictions for FRET have been observed, thus validated the theory, and the utility of this approach has stood the test of time [2], [26], [27], [28], [29], [30], [31]. It is less clear if we can simply assume that members of the green fluorescent protein (GFP) family [32], such as Cerulean and Venus, will behave like ‘typical’ fluorophores. DsRed is a red fluorescent protein structurally related to GFP [33], thought to form a complex of four closely associated fluorophores each enclosed in their β-barrel protein shells [34]. Single molecule bleaching experiments [35], [36], as well as a comparison of circular dichroism and absorption spectroscopy [37] both suggest the existence of excitonic behavior in this tetrameric DsRed assembly. As Förster remarked [1], under these conditions the *“excitation process itself is essentially shared with the neighboring molecules, and it is more correct to attribute the excitation energy to the whole system of molecules than to individual molecules.”* Essentially, donors and acceptors would behave as a single entity, not as individual fluorophores. If excitonic behavior were occurring in VCV or VCVV, FRET theory and the kinetic formalism for energy transfer to multiple acceptors would not be valid. As mentioned previously, excitonic behavior, or even weak-coupling is not thought to occur at physiological temperatures, particularly for fluorophores that are prevented from approaching each other as a result of steric hindrance [32]. While such an unorthodox quantum mechanical transfer mechanism seems unlikely in a biological context [38], the absence of other explanations compels us to speculate that perhaps the β-barrel structure of fluorescent proteins has been evolutionarily selected to allow either excitonic behavior, weak-coupling excitation, or an additional energy transfer path.

Regardless of the actual reasons for the higher amounts of energy transfer we have observed in constructs having multiple Venus FRET acceptors, our study indicates 1. That care must be taken when interpreting quantitative FRET experiments where multiple FRET acceptors might be present, and 2. The use of our experimental system, where we can design proteins with any arbitrary number of donors and acceptors, can be used to create higher efficiency pathways for energy transfer. This in turn can be used to optimize both energy trapping and funneling in nanotechnology applications. We speculate that the β-barrel structure of fluorescent proteins might be responsible for higher transfer rates when multiple acceptors are present. Even the remote possibility that fluorescent proteins under physiological conditions can transfer energy outside the Förster regime, indicates that further experiments are warranted.

## Materials and Methods

### Clone Construction

Restriction endonucleases were obtained from New England Biolabs (NEB, USA) or Roche (USA). *Pfu* Ultra (Stratagene, USA) was used in all polymerase chain reactions (PCR). All spectral variants of green fluorescent protein (FP's) [39] used in this study contained the A_206_K monomeric mutation [25]. Cloning and construction of Cerulean C1; a cyan FP with a single exponential lifetime decay [9], Venus C1; a yellow FP that folds rapidly [10], 6× His tagged Cerulean, 6× His tagged Venus, Cerulean-5-Venus (C5V), as well as the hetero-trimeric FP construct VCV; Venus-5-Cerulean-6-Venus (where the numbers indicate the number of amino acids separating the fluorophores) are described elsewhere as indicated [11], [16]. Amber C1; a Venus-“like” FP lacking a chromophore as a result of a single point mutation (Y_67_C) was generated by mutating the Tyrosine_67_ of Venus-C1 to Cysteine by using the sense primer 5′-ACCCTCGTGACCACCCTCGGCTGCGGCCTGCAGTGCTTCGCCCGC-3′ and the anti-sense primer 5′-GCGGGCGAAGCACTGCAGGCCGCAGCCGAGGGTGGTCACGAGGGT-3′. The resultant Amber C1 clone was confirmed by sequencing. The dual FP constructs C5A, V5A, A5C, V5C, V5V, A5V and A5A were constructed by first amplifying either Amber, Cerulean or Venus cDNA without the start codon using a sense primer with a BglII site (underlined) 5′-GCAGATCTGTGAGCAAGGGCGAGGAGCTGTTCACC-3′ and an anti sense primer with an EcoRI site (underlined) site 5′-GCGAATTCCTTGTACAGCTCGTCCATGCCGAGAGTG-3′ from either monomeric Amber-C1, Cerulean-C1or Venus-C1 vectors. The resultant fragments were cloned into Zero Blunt II Topo (Invitrogen, USA) and sequenced. Full-length cDNA for Amber was excised using BglII and EcoRI and cloned into Cerulean C1 or Venus C1 to generate C5A and V5A respectively. Similarly full length Cerulean cDNA was cloned into the BglII/EcoRI site of Amber C1 to generate A5C, or into Venus C1 to generate V5C. Full-length or Venus cDNA was cloned into BglII/EcoRI site of Amber C1 to generate A5V and Venus C1 to generate V5V. Constructs were validated by size using restriction digests. Full length Amber cDNA was cloned into BglII/EcoRI site of Amber C1 to generate A5A.

To generate the hetero-trimeric FP constructs: Amber-5-Cerulean-6-Amber (ACA), Venus-5-Cerulean-6-Amber (VCA), Amber-5-Cerulean-6-Venus (ACV), Amber-5-Venus-6-Amber (AVA), Venus-5-Amber-6-Amber (VAA), Amber-5-Amber-6-Venus (AAV) and Venus-5-Venus-6-Venus the Amber, Cerulean or Venus open reading frame (ORF) was amplified using a sense primer with a SalI site (underlined) 5′-GCGTCGACGGGTGAGCAAGGGCGAGGAGCTGTTCACCG-3′ and an anti-sense primer with a BamHI site (underlined) 5′-AGTCTCGGATCCCTTGTACAGCTCGTCCATGCCGAGAGTGATC-3. The resulting fragment was cloned and sequenced as described earlier. The Venus fragment was cloned into the SalI/BamHI digested AC to generate ACV, AA to generate AAV and VV to generate VVV. The Amber ORF was similarly cloned into VC to generate VCA and AC to generate ACA.

To generate the hetero-tetrameric FP contructs: Venus-5-Cerulean-5-Venus-6-Venus (VCVV), Amber-5-Cerulean-5-Venus-6-Amber (ACVA), Amber-5-Cerulean-5-Amber-6-Venus (ACAV) and Venus-5-Cerulean-5-Amber-6-Amber (VCAA) were constructed from VVV, AVA, VAA and AAA respectively. A sense primer with the BspE1 site (underlined) 5′-AGTCTCCGGAGGAGGTGGAAGCAAGGGCGAGGAGCTG-3′ and an anti sense primer with a BglII site (underlined) 5′-AGTCAGATCTTCCACCTCCCTTGTACAGCTCGTCCATGCC-3′ were used to amplify Cerulean from the Cerulean C1 vector. The DNA fragment was cloned into Zero Blunt II-TOPO, and the insert was sequenced. The insert was excised with BspE1 and BglII and cloned into VVV to generate VCVV, into AAV to generate ACAV, into VAA to generate VCAA, and into AVA to generate ACVA.

### Cell Culture and Transfection

HEK 293 cells (ATCC, USA) were cultured as a monolayer in a T-75 Flask (Corning, USA) in a humidified atmosphere containing 5% CO_2_ in air at 37°C in media containing DMEM with Hi glucose, sodium pyruvate, 10% fetal bovine serum, 1× NEAA, 1× Pen-Strep and 1× Glutamax (all purchased from Invitrogen, USA). Two days prior to imaging the cells were resuspended using TrypLE Express (Invitrogen, USA) and plated on 35 mm glass bottom dishes (Fluorodish, World Precision Instruments, USA). On the following day, 1 mg of plasmid cDNA was transfected into the cells using Lipofectamine 2000 (Invitrogen, USA) and incubated overnight and imaging was performed the following day in PBS (Media Tech, USA).

### Multi-Photon Microscopy

A mode locked Ti:sapphire laser (Coherent Chameleon, USA), running at 80 MHz, and tunable from 710–950 nm was attached to an upright Zeiss Axioplan-2 microscope with a Zeiss 510 META/NLO scan head and was used to acquire spectral images with two-photon excitation [40] for sRET analysis [16], [17]. After blocking excitation light using a BG39 filter placed in the light path spectral images with all 32 channels of the internal META detector were used to obtain emission spectra (spanning the range of 388–719 nm).

### sRET Analysis

Spectral images for sRET analysis was acquired as described earlier [16]. Briefly, pairs of spectral images of 1. capillaries containing 7.8 µM Cerulean or Venus, and 2. Cells transfected with a specific FRET construct, were collected with 860 and 900 nm excitation using a 20× NA 0.5 water objective. This set of 4 spectral images were then loaded into Igor Pro (Wavemetrics, USA), and processed with a macro that implements the sRET algorithm. FRET efficiencies from individual cells were measured. Average FRET efficiencies and statistical analysis were performed using Prism 5.0 (GraphPad software Inc., USA).

### E-FRET Analysis

E-FRET measurements were performed as described elsewhere [19]. Briefly, an IX-71 inverted microscope (Olympus , Japan) equipped with a 75 W Xenon arc lamp, a UNIBLITZ mechanical shutter (Vincent Associates, USA), a 60× oil objective (NA 1.4), a donor filter set (IDD cube, the donor channel; excitation: 436±10 nm, emitter: 480±20 nm, dichroic: 455LP), an acceptor filter set (IAA cube, the acceptor channel; excitation: 500±10 nm, emitter: 540±15 nm, dichroic: 520LP), and a FRET filter set (IDA cube, the FRET channel; excitation: 436±10 nm, emitter: 540±15 nm, dichroic: 455LP). A 12-bit cooled CCD camera (Retiga Exi, Qimaging, Canada) was used for data acquisition. The FRET efficiency and linear regression analysis of intermolecular FRET was calculated using Igor Pro (Wavemetrics) and statistical analysis was performed using Graph Pad Prism 5.0.

### Fluorescence Lifetime Microscopy

Fluorescence lifetime decay analysis was performed using time-correlated single-photon counting [12], as described earlier [11], [16]. A mode-locked laser tuned to 820 nm was used to excite Cerulean in constructs. Emitted photons were filtered through a BG39 filter, a 460–490 nm bandpass filter, a polarizer set to magic-angle conditions (54.7°, Medowlark Optics, USA), a 700 nm short pass filter (e700sp-2p; Chroma Optical, USA) and detected on a micro-channel plate photomultiplier (R3809U-52; Hamamatsu, Japan) attached to a Zeiss 510 non-descanned detector port placed in the transmitted light pathway. Photons were counted and correlated with excitation laser pulses using a SPC830 module (Becker and Hickl, Germany). Fluorescence lifetime decay curves were collected and processed as described earlier [11], [16]. Curves were dark count corrected. A second harmonic signal generated from sodium phosphate monobasic crystals irradiated with 940 nm light was used to measure the Instrument Response Function (IRF) of our system. As expected for a signal generated from a sub 200 femtosecond mode-locked laser, and a MCP-detector the measured full width at half maximal response was less than 40 ps. This Measured IRF was also used by SPCImage (Becker and Hickle Gmbh, Germany) to more accurately calculate the average lifetime decay of ACA (using a double exponential model) and Fluorescein (using a single exponential model).

### Emission Spectra

Spectral images of cells transfected with either Cerulean, Cerulean-Amber, or Cerulean-Venus were acquired on a Zeiss 510 META/NLO laser scanning microscope using a 40× NA 0.8 water objective. Spectra from three different cells were loaded into Igor Pro (Wavemetrics, USA). After subtracting background, each spectrum was normalized to the Cerulean peak at 481 nm and averaged.

### Multi-Photon Fluorescence Anisotropy Decay Measurements

Anisotropy decay measurements were performed as described earlier [41]. Briefly, a Zeiss 510 META/NLO laser scanning microscope modified for time-correlated single-photon counting [12] was used to acquire time-resolved anisotropy decay measurements. Transfected cells were imaged using a 40× NA 0.8 water objective. Constructs were excited with a mode-locked laser tuned to 950 nm. Emitted photons were filtered through a BG39 filter, a 535±15 nm band pass filter, a 700 nm short pass filter (Chroma Optical, USA), a polarizing beam splitter cube (Linos AG, Germany) augmented by two linear polarizers (Medowlark Optics, USA) mounted in each emission path from the cube splitter, and detected on two micro-channel plate photomultipliers (Hamamatsu R3809U-52, Japan) positioned on the parallel (I_VV_) and perpendicular (I_VH_) oriented polarization paths. Photons detected with the parallel or perpendicular detectors were multiplexed using a HR-41 four channel router (Becker and Hickl, Germany), and counted (and correlated with excitation laser pulses) using a SPC830 module (Becker and Hickl, Germany). Time resolved fluorescence anisotropy decay curves were generated from fluorescence lifetime decay curves generated from the photons detected with each photomultiplier using the following equation [13] for anisotropy (*r*):
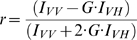

*G* is a microscope specific constant that accounts for different efficiencies for detecting photons in the *I_VV_* and *I_VH_* pathways. *G* was measured by tail fitting *I_VV_* and *I_VH_* lifetime decay curves of fluorescein, a sample known to rapidly depolarize [42], and was found to be ∼1.2.

### FRET Transfer Rate and Error Calculations

FRET rates (*k_D→A_*) were calculated from individual FRET efficiencies, as determined by E-FRET analysis, using the following equation:
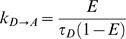
where *τ_D_* is the lifetime of the ACA construct (2.95 ns). Errors in these kinetic rates were calculated using error propagation [43] with Maple Software (Maplesoft, Waterloo, Canada):
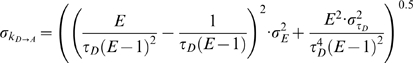
where 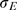 is the measured error in FRET efficiency measurements, and 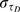 is the measured error in the lifetime of the donor in the absence of acceptors.

### Kinetic Model Predictions and Error Propagation

The kinetic model with multiple acceptors (eq. 2) can be expressed in terms of the observable FRET efficiencies, *E_i_*, for energy transfer from a single donor to acceptor *i* as:
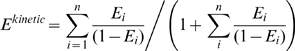
where *n* = 2, 3 when two or three acceptors are present. Note that the lifetime of the donor, *τ_D_*, is absent from this equation and therefore the donor lifetime (as well as the uncertainty of its value) does not effect the value or error in an ensemble FRET efficiency prediction.

The error in the predicted ensemble FRET efficiency using the kinetic model, 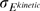, were derived using error propagation [43] with Maple Software (Maplesoft, Waterloo, Canada). The error in the FRET efficiency prediction for VCV is:
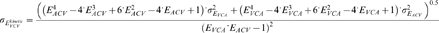



The calculation used to estimate the error in the FRET efficiency prediction for a donor with three acceptors, such as for VCVV, is considerably more complicated than the calculation for a donor with only two acceptors:
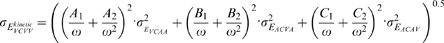
where the following substitutions are made:
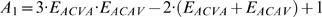


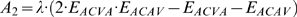


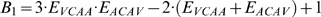


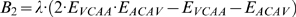


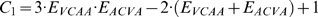


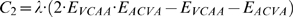


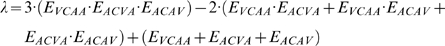


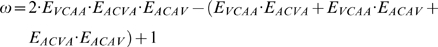



### Kinetic Model Analysis

Estimates for both 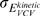
and 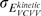 were calculated using the measured standard errors of the mean as estimates of 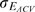, 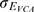, 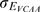, 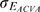 and 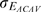. The differences between 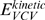
and 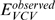 or between 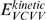 and 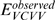 were calculated with error estimates. The difference 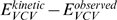 and 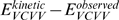 were both significantly different (p = 0.01) from zero. Error propagation calculations and statistical analysis were performed with Maple Software (Maplesoft, Waterloo, Canada).
